# Trial Sequential Analysis Comparing Bleeding and Major Adverse Cardiovascular Events in Patients with Atrial Fibrillation and Acute Coronary Syndrome on Dual versus Triple Therapy

**DOI:** 10.7759/cureus.4880

**Published:** 2019-06-11

**Authors:** Muhamad B Munir, Khansa Osman, Maryam Saleem, Kinjan Patel, Sudarshan Balla

**Affiliations:** 1 Cardiology, West Virginia University, J.W. Ruby Memorial Hospital, Morgantown, USA; 2 Internal Medicine, West Virginia University, J.W. Ruby Memorial Hospital, Morgantown, USA

**Keywords:** dual therapy, triple therapy, meta-analysis, atrial fibrillation, acute coronary syndrome

## Abstract

Objective

To assess efficacy and safety of dual therapy (DT) and triple therapy (TT) in patients with atrial fibrillation (AF) and acute coronary syndrome (ACS) with or without percutaneous coronary intervention (PCI) and evaluate the quality of evidence with respect to said outcomes based on contemporary randomized trials (RCTs). The efficacy outcome taken was major adverse cardiovascular events (MACE) while safety outcome was major bleeding events.

Introduction

Appropriate anti-thrombotic therapy is still controversial in patients with AF and concomitant ACS or PCI. We conducted a conventional meta-analysis pooling data from major RCTs to assess the efficacy and safety of DT and TT. Additionally, we utilized advanced analytic properties of trial sequential analysis (TSA) to assess for quality of evidence in this realm.

Methods and results

A total of 8,732 patients from five major RCTs were enrolled in this study. There was a statistically significant reduction in major bleeding on the DT group compared to the TT group (RR 0.65, 95% CI 0.48, 0.86). The incidence of major adverse cardiovascular events (MACE) was similar in both groups (RR 0.97, 95% CI 0.8,1.17). The trial sequential analysis showed strong evidence supporting reduction in bleeding from current major RCTs while being inconclusive based on MACE outcome.

Conclusion

Sufficient quality evidence could be ascertained from contemporary RCTs on reduced incidence of bleeding in DT patients compared to TT patients. Further adequately powered RCTs are needed to ensure non-inferiority of DT over TT with respect to MACE outcome.

## Introduction

The management of patients with atrial fibrillation (AF) and acute coronary syndrome (ACS) or percutaneous coronary intervention (PCI) continues to be challenging in term of antithrombotic therapy choice. Triple therapy (TT) with an oral anticoagulant and dual antiplatelet medications is currently endorsed as the therapy of choice by the European guidelines in this patient population [1]. In contrast, North American guidelines recommend dual therapy (DT) with new oral anticoagulant and P2Y_12_ inhibitor [2]. 

We used the advanced meta-analytic properties of trial sequential analysis (TSA) to assess the quality of available evidence comparing TT vs. DT from current major randomized controlled trials (RCTs). For the purpose of our analysis, we used major adverse cardiovascular events (MACE) as an efficacy outcome while major bleeding was taken as a safety outcome.

## Materials and methods

For the current study, data was pooled from five major RCTs that compared DT and TT in AF patients with associated ACS and/or PCI. The RCTs used to collect data for our current analysis included the recently published Open-label, 2x2 Factorial, Randomized Controlled, Clinical Trial to Evaluate the Safety of Apixaban vs. Vitamin K Antagonist and Aspirin vs. Aspirin Placebo in Patients with Atrial Fibrillation and Acute Coronary Syndrome or Percutaneous Coronary Intervention (AUGUSTUS) trial [3] and previously published Randomized Evaluation of Dual Antithrombotic Therapy With Dabigatran vs Triple Therapy With Warfarin in Patients With Nonvalvular Atrial Fibrillation Undergoing Percutaneous Coronary Intervention (RE‐DUAL PCI) trial [4], Open-Label, Randomized, Controlled, Multicenter Study Exploring Two Treatment Strategies of Rivaroxaban and a Dose-Adjusted Oral Vitamin K Antagonist Treatment Strategy in Subjects with Atrial Fibrillation who Undergo Percutaneous Coronary Intervention (PIONEER-AF PCI) trial [5], Intracoronary Stenting and Antithrombotic Regimen-Testing of a 6-Week Versus a 6-Month Clopidogrel Treatment Regimen in Patients With Concomitant Aspirin and Oral Anticoagulant Therapy Following Drug-Eluting Stenting (ISAR-TRIPLE) trial [6], and What is the Optimal Antiplatelet and anticoagulant therapy in patients with oral anticoagulation and coronary StenTing (WOEST) trials [7]. The relevant data was collected into Microsoft Excel worksheet. For the purpose of our analysis, we extracted data from patients on 150 mg of dabigatran twice a day from RE-DUAL PCI trial and on 15 mg rivaroxaban daily from PIONEER AF trial. Since our study contained pooled patient data from these RCTs, the need for institutional review board was deferred.

TSA can be applied to quantify the reliability of conclusions driven from meta-analysis by establishing monitoring boundaries to test the quality of evidence. By this method, if the cumulative Z curve crossed the TSA boundary, a sufficient level of evidence has been reached supporting the intervention. However, if the Z curve failed to cross the TSA boundary, evidence to reach a conclusion is insufficient and more studies are needed. We pooled the primary safety outcome of bleeding (defined as Thrombolysis in Myocardial Infarction major and minor bleeding) and the primary efficacy outcome of major adverse cardiovascular events (composite of cardiac death, stent thrombosis, stroke and myocardial infarction) using the random effect model from above RCTs comparing DT to TT at the maximum reported follow-up. We then performed TSA to maintain an overall two-sided type-I error rate at 5% and calculated the required sample size to achieve 80% power to detect a statistically significant difference. The analysis was conducted using RevMan 5.3 (The Cochrane Collaboration, The Nordic Cochrane Centre, Copenhagen, Denmark) and Copenhagen Trial Unit, version 0.9.5.10 beta.

Limitations

Few limitations of our study should be acknowledged. We do not have patient-level data for our current meta-analysis and utilized trial-level data so we cannot account for differences in baseline characteristics that may affect outcomes. Additionally, differential trial designs may have instituted heterogeneity although we tried to mitigate this by depicting statistical heterogeneity for each outcome studied.

## Results

**Figure 1:**
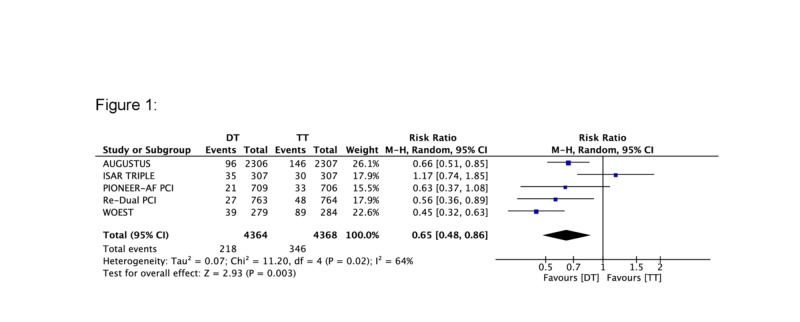
Forest plot comparing bleeding events between triple therapy (TT) and dual therapy (DT)

**Figure 2:**
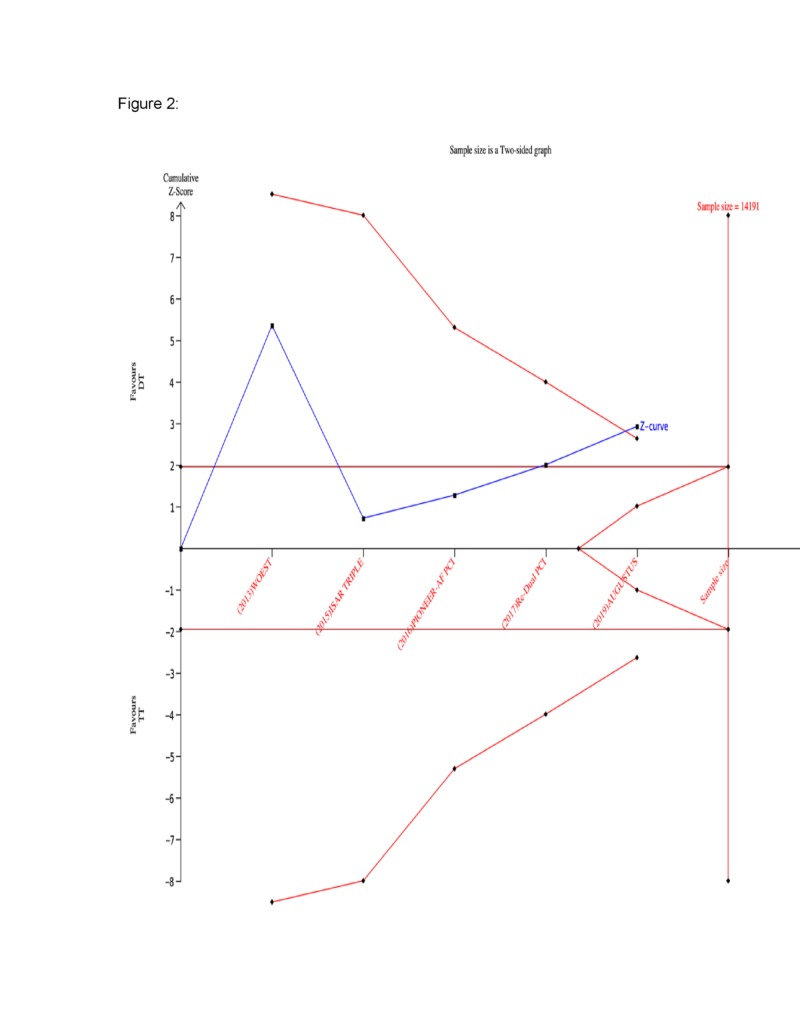
Trial sequential analysis for bleeding events The cumulative Z-curve (blue line with small black squares representing each trial) crosses both the traditional (horizontal red line) and the trial sequential monitoring boundary (concave red line), indicating firm evidence of better outcomes in the dual therapy group compared to the triple therapy.

**Figure 3:**
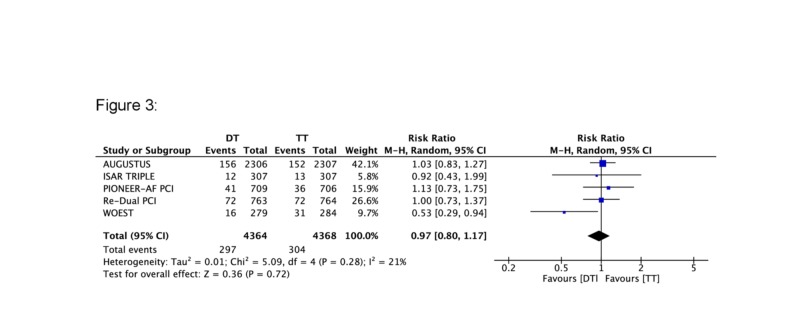
Forest plot comparing major adverse cardiovascular events (MACE) between triple therapy (TT) and dual therapy (DT)

**Figure 4:**
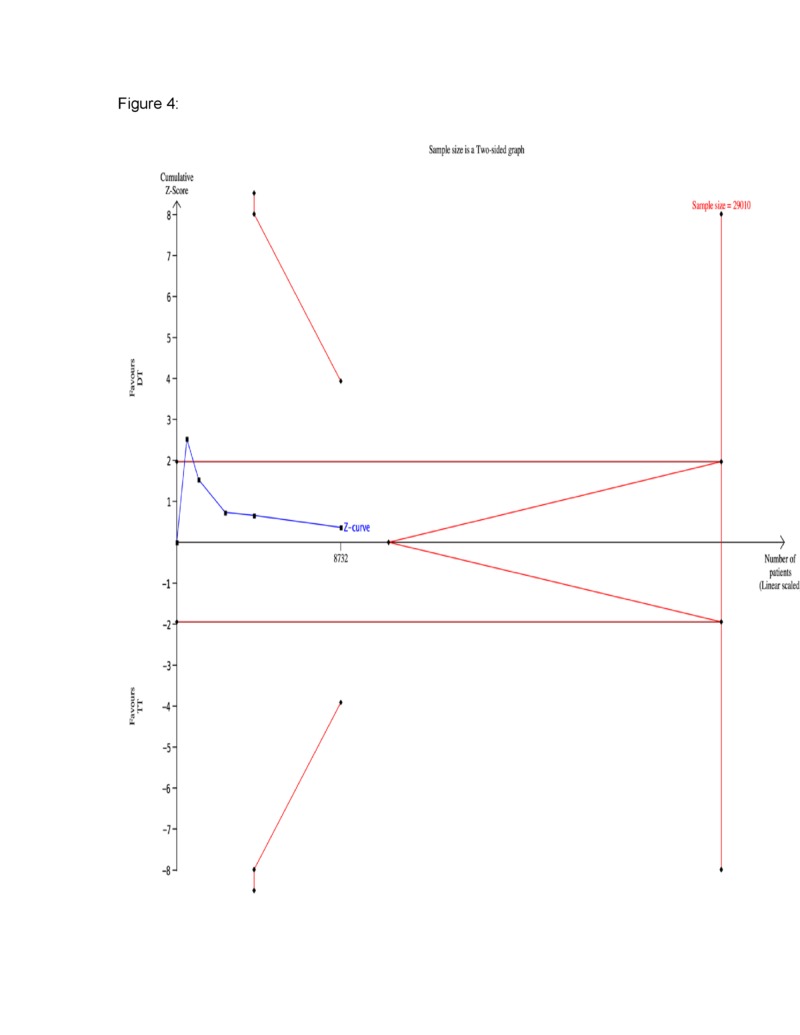
Trial sequential analysis for major adverse cardiovascular events (MACE) The cumulative Z-curve (blue line with small black squares representing each trial) failed to cross both the traditional (horizontal red line) and the trial sequential monitoring boundary (concave red line), indicating no difference between both groups and no sufficient evidence to meet the trial sequential analysis (TSA) boundary.

A total of five major RCTs with 8,732 patients were included in the current analysis. A statistically significant reduction in the rate of bleeding was seen in the DT group compared to the TT group (RR 0.65, 95% CI 0.48, 0.86) (Figure 1). The corresponding Z-curve successfully crossed the conventional test boundary as well as the TSA monitoring boundary indicating firm evidence supporting the lower rate of bleeding in DT (Figure 2). On the other hand, there was no difference in MACE between the two groups (RR 0.97, 95% CI 0.8,1.17) (Figure 3). The MACE Z-curve failed to cross the conventional and TSA test boundaries indicating that no firm conclusion could be derived on the benefit of TT over DT in preventing MACE outcomes in these patients (Figure 4).

## Discussion

The findings from our current analysis indicate firm evidence supporting lower rates of bleeding in patients treated with DT vs TT. Our conventional meta-analysis further showed that TT confers no additional benefit in preventing MACE outcomes when compared to DT. TSA analysis, however, concluded that sufficient evidence could not be obtained with respect to MACE outcomes among patients on DT and TT. The management of AF patients with concomitant ACS or PCI is fraught with uncertainty. While oral anticoagulants are shown to be superior to dual anti-platelet agents (DAPT) in reducing risk of ischemic stroke and systemic embolism in AF patients, they don’t confer any benefit in preventing complications such as stent thrombosis [8]. On the contrary, DAPT has shown to have superior efficacy in preventing cardiovascular ischemic events and stent thrombosis but have not shown any benefit in reducing embolic events in AF patients. The usual contemporary practice is to place these patients on triple therapy which make them prone to significant bleeding complications. The increased benefit of preventing MACE outcomes may be meager while on triple therapy. Our analysis has shown a significant benefit of DT versus TT in reducing bleeding complications with TSA endorsing sufficient evidence from current trials in supporting this conclusion. On the contrary, our conventional meta-analysis did not show any statistically significant reduction in MACE events with TT when compared to DT (RR 0.97, 95% CI 0.8,1.17). It is pertinent to point out that advanced meta-analytical model of TSA concluded that no firm evidence could be obtained with respect to MACE outcomes based on patients utilization of DT or TT.

## Conclusions

Our conventional meta-analysis showed a significantly improved rate of bleeding in AF patients with concomitant ACS and/or PCI on DT compared to TT while at the same time showed similar incidence of MACE outcomes in both groups. TSA confirmed that sufficient quality evidence exists from current RCTs that proves low incidence of bleeding in the DT group compared to the TT group. However, more adequately powered RCTs are required to ensure non-inferiority of DT over TT in preventing MACE related outcomes.

## Appendices

Both Drs. Munir and Osman contributed equally to this work. 
